# Poly[[triaqua­(μ_3_-4-oxidopyridine-2,6-dicarboxyl­ato)holmium(III)] mono­hydrate]

**DOI:** 10.1107/S1600536811016953

**Published:** 2011-05-11

**Authors:** Zhu-Qing Gao, Dong-Yu Lv, Jin-Zhong Gu, Hong-Jin Li

**Affiliations:** a School of Chemistry and Biological Engineering, Taiyuan University of Science and Technology, Taiyuan 030021, People’s Republic of China; b Key Laboratory of Nonferrous Metal Chemistry and Resources Utilization of Gansu Province, College of Chemistry and Chemical Engineering, Lanzhou University, Lanzhou 730000, People’s Republic of China

## Abstract

In the title coordination polymer, {[Ho(C_7_H_2_NO_5_)(H_2_O)_3_]·H_2_O}_*n*_, the Ho^III^ atom is eight-coordinated by a tridentate 4-oxidopyridine-2,6-dicarboxyl­ate trianion, two monodentate anions and three water mol­ecules, forming a distorted bicapped trigonal–prismatic HoNO_7_ coordination geometry. The anions bridge adjacent Ho^III^ ions into double chains. Adjacent chains are further connected into sheets. O—H⋯O hydrogen bonds involving both coordinated and uncoordinated water mol­ecules generate a three-dimensional supra­molecular framework.

## Related literature

For the structures and properties of lanthanide coordination compounds, see: Wang *et al.* (2007 ▶); Lv *et al.* (2010 ▶); Gao *et al.* (2006 ▶). For bond lengths and angles in other complexes with eight-coordinate Ho^III^, see: Wang *et al.* (2007 ▶); Munoz *et al.* (2005 ▶).
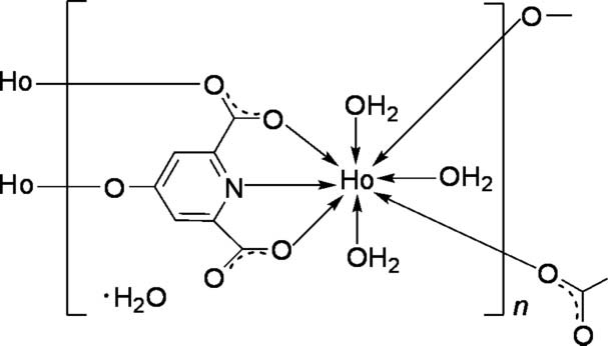


## Experimental

### 

#### Crystal data

[Ho(C_7_H_2_NO_5_)(H_2_O)_3_]·H_2_O
*M* *_r_* = 417.09
Monoclinic, 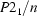

*a* = 9.879 (5) Å
*b* = 7.557 (4) Å
*c* = 15.386 (8) Å
β = 105.386 (5)°
*V* = 1107.5 (9) Å^3^
*Z* = 4
Mo *K*α radiation
μ = 7.19 mm^−1^
*T* = 296 K
0.32 × 0.29 × 0.26 mm

#### Data collection

Bruker APEXII CCD diffractometer
Absorption correction: multi-scan (*SADABS*; Bruker, 2004 ▶) *T*_min_ = 0.207, *T*_max_ = 0.257
4776 measured reflections
2003 independent reflections
1646 reflections with *I* > 2σ(*I*)
*R*_int_ = 0.025

#### Refinement

*R*[*F* ^2^ > 2σ(*F* ^2^)] = 0.021
*wR*(*F* ^2^) = 0.047
*S* = 1.02
2003 reflections
196 parameters
12 restraints
H atoms treated by a mixture of independent and constrained refinement
Δρ_max_ = 0.71 e Å^−3^
Δρ_min_ = −0.54 e Å^−3^

### 

Data collection: *APEX2* (Bruker, 2004 ▶); cell refinement: *SAINT* (Bruker, 2004 ▶); data reduction: *SAINT*; program(s) used to solve structure: *SHELXS97* (Sheldrick, 2008 ▶); program(s) used to refine structure: *SHELXL97* (Sheldrick, 2008 ▶); molecular graphics: *SHELXTL* (Sheldrick, 2008 ▶); software used to prepare material for publication: *SHELXTL*.

## Supplementary Material

Crystal structure: contains datablocks I, global. DOI: 10.1107/S1600536811016953/sj5136sup1.cif

Structure factors: contains datablocks I. DOI: 10.1107/S1600536811016953/sj5136Isup2.hkl

Additional supplementary materials:  crystallographic information; 3D view; checkCIF report

## Figures and Tables

**Table 1:** Hydrogen-bond geometry (Å, °)

*D*—H⋯*A*	*D*—H	H⋯*A*	*D*⋯*A*	*D*—H⋯*A*
O9—H8*W*⋯O2^i^	0.86 (1)	2.33 (4)	3.080 (5)	145 (6)
O9—H7*W*⋯O4	0.86 (1)	1.84 (1)	2.693 (5)	170 (5)
O8—H6*W*⋯O9^ii^	0.86 (1)	2.28 (3)	3.026 (5)	145 (5)
O8—H5*W*⋯O9^iii^	0.86 (1)	1.84 (2)	2.689 (5)	168 (5)
O7—H4*W*⋯O3^iii^	0.86 (1)	2.08 (3)	2.788 (4)	139 (4)
O7—H3*W*⋯O5^iv^	0.87 (1)	1.92 (3)	2.711 (4)	152 (4)
O6—H2*W*⋯O1^v^	0.86 (1)	1.85 (2)	2.671 (4)	159 (5)
O6—H1*W*⋯O4^vi^	0.86 (1)	1.83 (1)	2.687 (4)	171 (5)

Symmetry codes: (i) 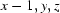; (ii) 
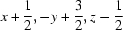
; (iii) 
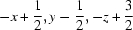
; (iv) 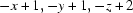; (v) 
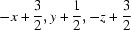
; (vi) 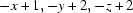.

**Fig. 1.**
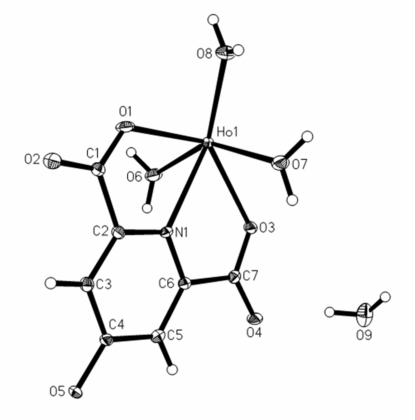
The asymmetric unit of the title complex, showing the atom-labeling scheme. Displacement ellipsoids are drawn at the 30% probability level.

**Fig. 2.**
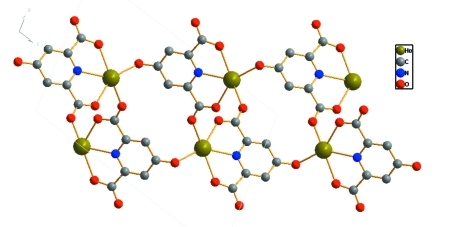
View along the b axis, showing the polymeric double chain.

**Fig. 3.**
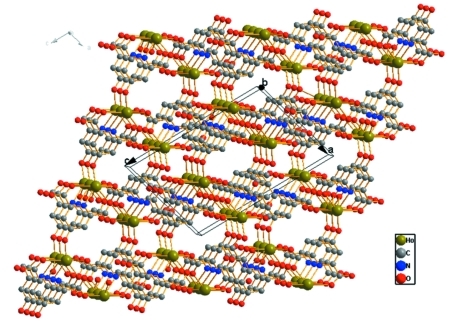
View along the a axis, showing the sheet structure of {[Ho(C7H2NO5)(H2O)3].H2O}n.

## supplementary crystallographic information

### Comment

Lanthanide coordination polymers have shown not only versatile architectures but also desirable properties, *e.g.*, luminescent, magnetic, catalytic, and gas absorption and separation properties (Wang *et al.*, 2007; Lv *et al.*, 2010). In order to extend our investigations in this field, we designed and synthesized one lanthanide coordination polymer {[Ho(C_7_H_2_NO_5_)(H_2_O)_3_].H_2_O}*_n_* by choosing 4-oxidopyridine-2,6-dicarboxylicacid as a functional ligand, and report its structure here.

The title compound is isotypic with its Dy (Gao *et al.*, 2006) and Eu (Lv *et al.*, 2010) analogues. As shown in Fig.1, the asymmetric unit of the cell contains one Ho(III) ion, one 4-oxidopyridine-2,6-dicarboxylate anion, three coordinated water molecules, and one water molecule of crystallization. The Ho atom is eight-coordinated by seven oxygen atoms from three anions and three coordinated water molecules and by one nitrogen atom from one tridentate anion (the other two anions are monodentate), forming a distorted bicapped trigonal-prismatic coordination environment.

Important bond distances are presented in Table 1. The Ho–O bond lengths [2.279 (3) to 2.400 (3) Å] are shorter than the Ho–N bond length [2.450 (3) Å], which is in agreement with the bond lengths observed in other Ho(III) complexes (Wang *et al.*, 2007; Munoz *et al.*, 2005). The anion adopts a *µ*_3_-pentadentate coordination mode, as shown in Fig.1. The anions bridge the adjacent Ho^III^ ions to form infinite double chains (Fig.2). Adjacent chains are further connected by the coordination of the anions and Ho(III) ions into a two-dimensional sheet (Fig.3), which are further extended into a three-dimensional supramolecular framework through O–H···O hydrogen-bonding interactions including both coordinated and uncoordinated water molecules (Table 2).

### Experimental

To a solution of holmium(III)nitrate hexahydrate (0.138 g, 0.3 mmol) in water (5 ml) was added an aqueous solution (5 ml) of the ligand (0.060 g, 0.3 mmol) and a drop of triethylamine. The reactants were sealed in a 25-ml Teflon-lined, stainless-steel Parr bomb. The bomb was heated at 433 K for 3 days. The cool solution yielded colorless single crystals in *ca* 75% yield. Anal. Calcd for C_7_H_10_TbNO_9_: C, 20.16; H, 2.42; N, 3.36. Found: C, 20.45; H, 2.17; N, 3.64.

### Refinement

The coordinated water H atoms were located in a different Fourier map and refined with distance constraints of O–H = 0.83 (3) Å. The free water H atoms were placed at calculated positions and refined with a riding model, considering the position of oxygen atoms and the quantity of H atoms. The carbon-bound H atoms were placed in geometrically idealized positions, with C–H = 0.93 Å and constrained to ride on their respective parent atoms, with *U*iso(H) = 1.2 *U*eq(C).

### Crystal data

**Table:**

[Ho(C_7_H_2_NO_5_)(H_2_O)_3_]·H_2_O	*F*(000) = 792
*M**_r_* = 417.09	*D*_x_ = 2.502 Mg m^−^^3^
Monoclinic, *P*2_1_/*n*	Mo *K*α radiation, λ = 0.71073 Å
Hall symbol: -P 2y/n	Cell parameters from 2292 reflections
*a* = 9.879 (5) Å	θ = 2.2–28.2°
*b* = 7.557 (4) Å	µ = 7.19 mm^−^^1^
*c* = 15.386 (8) Å	*T* = 296 K
β = 105.386 (5)°	Block, colorless
*V* = 1107.5 (9) Å^3^	0.32 × 0.29 × 0.26 mm
*Z* = 4	

### Data collection

**Table:**

Bruker APEXII CCD diffractometer	2003 independent reflections
Radiation source: fine-focus sealed tube	1646 reflections with *I* > 2σ(*I*)
graphite	*R*_int_ = 0.025
φ and ω scans	θ_max_ = 25.5°, θ_min_ = 2.2°
Absorption correction: multi-scan (*SADABS*; Bruker, 2004)	*h* = −11→7
*T*_min_ = 0.207, *T*_max_ = 0.257	*k* = −8→9
4776 measured reflections	*l* = −16→17

### Refinement

**Table:**

Refinement on *F*^2^	Secondary atom site location: difference Fourier map
Least-squares matrix: full	Hydrogen site location: inferred from neighbouring sites
*R*[*F*^2^ > 2σ(*F*^2^)] = 0.021	H atoms treated by a mixture of independent and constrained refinement
*wR*(*F*^2^) = 0.047	*w* = 1/[σ^2^(*F*_o_^2^) + (0.0176*P*)^2^] where *P* = (*F*_o_^2^ + 2*F*_c_^2^)/3
*S* = 1.02	(Δ/σ)_max_ = 0.002
2003 reflections	Δρ_max_ = 0.71 e Å^−^^3^
196 parameters	Δρ_min_ = −0.54 e Å^−^^3^
12 restraints	Extinction correction: *SHELXL97* (Sheldrick, 2008), Fc^*^=kFc[1+0.001xFc^2^λ^3^/sin(2θ)]^-1/4^
Primary atom site location: structure-invariant direct methods	Extinction coefficient: 0.0119 (3)

### Special details

**Table:**

Geometry. All e.s.d.'s (except the e.s.d. in the dihedral angle between two l.s. planes) are estimated using the full covariance matrix. The cell e.s.d.'s are taken into account individually in the estimation of e.s.d.'s in distances, angles and torsion angles; correlations between e.s.d.'s in cell parameters are only used when they are defined by crystal symmetry. An approximate (isotropic) treatment of cell e.s.d.'s is used for estimating e.s.d.'s involving l.s. planes.
Refinement. Refinement of *F*^2^ against ALL reflections. The weighted *R*-factor *wR* and goodness of fit *S* are based on *F*^2^, conventional *R*-factors *R* are based on *F*, with *F* set to zero for negative *F*^2^. The threshold expression of *F*^2^ > σ(*F*^2^) is used only for calculating *R*-factors(gt) *etc*. and is not relevant to the choice of reflections for refinement. *R*-factors based on *F*^2^ are statistically about twice as large as those based on *F*, and *R*- factors based on ALL data will be even larger.

### Fractional atomic coordinates and isotropic or equivalent isotropic displacement parameters (Å^2^)

**Table:**

	*x*	*y*	*z*	*U*_iso_*/*U*_eq_	
Ho1	0.501337 (17)	0.82185 (2)	0.747304 (11)	0.01332 (10)	
C1	0.7829 (4)	0.5891 (5)	0.8379 (3)	0.0184 (9)	
C2	0.7250 (4)	0.6133 (5)	0.9175 (3)	0.0158 (9)	
C3	0.7940 (4)	0.5582 (5)	1.0018 (3)	0.0172 (9)	
H3	0.8780	0.4963	1.0112	0.021*	
C4	0.7379 (4)	0.5951 (5)	1.0743 (2)	0.0163 (9)	
C5	0.6087 (4)	0.6828 (5)	1.0537 (3)	0.0193 (9)	
H5	0.5652	0.7073	1.0991	0.023*	
C6	0.5460 (4)	0.7328 (5)	0.9665 (3)	0.0157 (9)	
C7	0.4083 (4)	0.8304 (5)	0.9374 (3)	0.0162 (9)	
H1W	0.613 (6)	1.087 (8)	0.8854 (11)	0.08 (2)*	
H2W	0.661 (5)	1.128 (7)	0.806 (3)	0.08 (2)*	
H3W	0.332 (5)	0.547 (8)	0.7874 (18)	0.09 (2)*	
H4W	0.313 (4)	0.523 (6)	0.6913 (15)	0.040 (15)*	
H5W	0.484 (6)	0.5513 (17)	0.606 (4)	0.08 (2)*	
H6W	0.524 (8)	0.714 (7)	0.569 (3)	0.12 (3)*	
H7W	0.1510 (15)	0.841 (6)	0.945 (3)	0.057 (19)*	
H8W	0.032 (5)	0.745 (8)	0.888 (4)	0.16 (4)*	
N1	0.6032 (3)	0.7004 (4)	0.8981 (2)	0.0151 (7)	
O1	0.7200 (3)	0.6723 (4)	0.76661 (19)	0.0274 (8)	
O2	0.8899 (3)	0.4962 (4)	0.84514 (18)	0.0234 (7)	
O3	0.3689 (3)	0.8732 (4)	0.85503 (18)	0.0201 (6)	
O4	0.3418 (3)	0.8625 (4)	0.99379 (18)	0.0249 (7)	
O5	0.8051 (3)	0.5493 (4)	1.15748 (17)	0.0200 (6)	
O6	0.6030 (3)	1.0722 (4)	0.8286 (2)	0.0315 (8)	
O7	0.3636 (3)	0.5656 (4)	0.7411 (2)	0.0295 (8)	
O8	0.4963 (4)	0.6642 (4)	0.6113 (2)	0.0255 (7)	
O9	0.0620 (4)	0.8221 (4)	0.9302 (3)	0.0392 (9)	

### Atomic displacement parameters (Å^2^)

**Table:**

	*U*^11^	*U*^22^	*U*^33^	*U*^12^	*U*^13^	*U*^23^
Ho1	0.01235 (13)	0.01734 (13)	0.01046 (14)	0.00009 (7)	0.00339 (8)	0.00057 (8)
C1	0.016 (2)	0.024 (2)	0.014 (2)	0.0019 (17)	0.0032 (17)	−0.0012 (17)
C2	0.015 (2)	0.018 (2)	0.014 (2)	0.0028 (16)	0.0033 (17)	−0.0011 (17)
C3	0.016 (2)	0.018 (2)	0.017 (2)	0.0040 (16)	0.0039 (17)	0.0012 (16)
C4	0.017 (2)	0.019 (2)	0.013 (2)	−0.0047 (16)	0.0028 (17)	0.0022 (17)
C5	0.019 (2)	0.022 (2)	0.019 (2)	−0.0011 (16)	0.0079 (18)	0.0022 (17)
C6	0.016 (2)	0.017 (2)	0.014 (2)	0.0005 (16)	0.0049 (17)	0.0004 (16)
C7	0.016 (2)	0.015 (2)	0.018 (2)	−0.0025 (15)	0.0059 (18)	−0.0032 (16)
N1	0.0145 (18)	0.0198 (19)	0.0126 (18)	0.0011 (13)	0.0063 (14)	0.0005 (13)
O1	0.0270 (17)	0.044 (2)	0.0138 (16)	0.0158 (14)	0.0108 (14)	0.0074 (13)
O2	0.0244 (17)	0.0273 (18)	0.0196 (16)	0.0151 (13)	0.0076 (13)	0.0013 (13)
O3	0.0150 (15)	0.0300 (17)	0.0150 (16)	0.0057 (12)	0.0033 (12)	0.0029 (12)
O4	0.0211 (16)	0.0410 (19)	0.0151 (16)	0.0065 (13)	0.0092 (13)	0.0003 (13)
O5	0.0158 (15)	0.0295 (17)	0.0124 (15)	−0.0036 (12)	−0.0005 (12)	0.0065 (12)
O6	0.036 (2)	0.041 (2)	0.021 (2)	−0.0177 (15)	0.0143 (16)	−0.0087 (15)
O7	0.0341 (19)	0.036 (2)	0.0202 (18)	−0.0165 (14)	0.0105 (16)	−0.0040 (15)
O8	0.036 (2)	0.0232 (19)	0.0186 (18)	−0.0012 (14)	0.0098 (15)	−0.0031 (13)
O9	0.027 (2)	0.029 (2)	0.059 (3)	−0.0001 (15)	0.0072 (19)	0.0033 (18)

### Geometric parameters (Å, °)

**Table:**

Ho1—O5^i^	2.279 (3)	C5—C6	1.373 (5)
Ho1—O6	2.342 (3)	C5—H5	0.9300
Ho1—O7	2.354 (3)	C6—N1	1.341 (5)
Ho1—O1	2.386 (3)	C6—C7	1.508 (5)
Ho1—O2^ii^	2.393 (3)	C7—O4	1.243 (5)
Ho1—O8	2.397 (3)	C7—O3	1.265 (5)
Ho1—O3	2.400 (3)	O2—Ho1^iii^	2.393 (3)
Ho1—N1	2.450 (3)	O5—Ho1^iv^	2.279 (3)
C1—O2	1.249 (5)	O6—H1W	0.860 (10)
C1—O1	1.275 (5)	O6—H2W	0.858 (10)
C1—C2	1.493 (5)	O7—H3W	0.865 (10)
C2—N1	1.334 (5)	O7—H4W	0.861 (10)
C2—C3	1.361 (5)	O8—H5W	0.862 (10)
C3—C4	1.399 (5)	O8—H6W	0.861 (10)
C3—H3	0.9300	O9—H7W	0.860 (10)
C4—O5	1.322 (4)	O9—H8W	0.861 (10)
C4—C5	1.398 (5)		
			
O5^i^—Ho1—O6	98.86 (11)	C3—C2—C1	122.7 (3)
O5^i^—Ho1—O7	86.39 (12)	C2—C3—C4	119.7 (4)
O6—Ho1—O7	147.96 (12)	C2—C3—H3	120.2
O5^i^—Ho1—O1	150.97 (10)	C4—C3—H3	120.2
O6—Ho1—O1	94.04 (12)	O5—C4—C5	122.5 (4)
O7—Ho1—O1	96.31 (12)	O5—C4—C3	121.1 (4)
O5^i^—Ho1—O2^ii^	81.27 (10)	C5—C4—C3	116.5 (4)
O6—Ho1—O2^ii^	71.09 (11)	C6—C5—C4	119.9 (4)
O7—Ho1—O2^ii^	140.78 (10)	C6—C5—H5	120.0
O1—Ho1—O2^ii^	78.49 (10)	C4—C5—H5	120.0
O5^i^—Ho1—O8	82.29 (11)	N1—C6—C5	122.8 (4)
O6—Ho1—O8	140.74 (12)	N1—C6—C7	113.0 (3)
O7—Ho1—O8	71.19 (12)	C5—C6—C7	124.2 (4)
O1—Ho1—O8	71.43 (11)	O4—C7—O3	124.8 (4)
O2^ii^—Ho1—O8	70.33 (11)	O4—C7—C6	119.4 (4)
O5^i^—Ho1—O3	79.50 (10)	O3—C7—C6	115.8 (3)
O6—Ho1—O3	74.68 (11)	C2—N1—C6	117.2 (3)
O7—Ho1—O3	75.28 (11)	C2—N1—Ho1	121.0 (3)
O1—Ho1—O3	129.20 (9)	C6—N1—Ho1	121.4 (2)
O2^ii^—Ho1—O3	137.27 (9)	C1—O1—Ho1	124.0 (2)
O8—Ho1—O3	142.60 (11)	C1—O2—Ho1^iii^	139.4 (3)
O5^i^—Ho1—N1	143.51 (10)	C7—O3—Ho1	124.8 (2)
O6—Ho1—N1	77.69 (12)	C4—O5—Ho1^iv^	127.1 (2)
O7—Ho1—N1	79.53 (11)	Ho1—O6—H1W	124 (3)
O1—Ho1—N1	64.77 (10)	Ho1—O6—H2W	115 (3)
O2^ii^—Ho1—N1	129.28 (10)	H1W—O6—H2W	116 (2)
O8—Ho1—N1	123.29 (11)	Ho1—O7—H3W	116 (3)
O3—Ho1—N1	64.44 (10)	Ho1—O7—H4W	123 (3)
O2—C1—O1	124.2 (4)	H3W—O7—H4W	114.8 (19)
O2—C1—C2	119.7 (3)	Ho1—O8—H5W	123 (3)
O1—C1—C2	116.1 (3)	Ho1—O8—H6W	120 (4)
N1—C2—C3	123.9 (4)	H5W—O8—H6W	115.3 (19)
N1—C2—C1	113.4 (3)	H7W—O9—H8W	116 (2)
			
O2—C1—C2—N1	−173.7 (4)	O3—Ho1—N1—C2	−179.5 (3)
O1—C1—C2—N1	8.7 (5)	O5^i^—Ho1—N1—C6	−15.3 (4)
O2—C1—C2—C3	9.1 (6)	O6—Ho1—N1—C6	72.9 (3)
O1—C1—C2—C3	−168.6 (4)	O7—Ho1—N1—C6	−84.4 (3)
N1—C2—C3—C4	−1.0 (6)	O1—Ho1—N1—C6	173.5 (3)
C1—C2—C3—C4	175.9 (4)	O2^ii^—Ho1—N1—C6	125.6 (3)
C2—C3—C4—O5	−177.4 (4)	O8—Ho1—N1—C6	−143.5 (3)
C2—C3—C4—C5	2.3 (6)	O3—Ho1—N1—C6	−5.8 (3)
O5—C4—C5—C6	177.7 (4)	O2—C1—O1—Ho1	172.6 (3)
C3—C4—C5—C6	−2.0 (6)	C2—C1—O1—Ho1	−9.9 (5)
C4—C5—C6—N1	0.3 (6)	O5^i^—Ho1—O1—C1	−163.5 (3)
C4—C5—C6—C7	−178.9 (4)	O6—Ho1—O1—C1	80.1 (3)
N1—C6—C7—O4	177.2 (3)	O7—Ho1—O1—C1	−69.5 (3)
C5—C6—C7—O4	−3.4 (6)	O2^ii^—Ho1—O1—C1	149.8 (3)
N1—C6—C7—O3	−2.4 (5)	O8—Ho1—O1—C1	−137.3 (3)
C5—C6—C7—O3	176.9 (4)	O3—Ho1—O1—C1	6.6 (4)
C3—C2—N1—C6	−0.8 (6)	N1—Ho1—O1—C1	5.7 (3)
C1—C2—N1—C6	−178.0 (3)	O1—C1—O2—Ho1^iii^	−28.4 (7)
C3—C2—N1—Ho1	173.2 (3)	C2—C1—O2—Ho1^iii^	154.1 (3)
C1—C2—N1—Ho1	−4.0 (5)	O4—C7—O3—Ho1	177.4 (3)
C5—C6—N1—C2	1.1 (6)	C6—C7—O3—Ho1	−3.0 (5)
C7—C6—N1—C2	−179.6 (3)	O5^i^—Ho1—O3—C7	178.8 (3)
C5—C6—N1—Ho1	−172.8 (3)	O6—Ho1—O3—C7	−78.9 (3)
C7—C6—N1—Ho1	6.5 (4)	O7—Ho1—O3—C7	89.8 (3)
O5^i^—Ho1—N1—C2	171.0 (3)	O1—Ho1—O3—C7	3.6 (3)
O6—Ho1—N1—C2	−100.7 (3)	O2^ii^—Ho1—O3—C7	−116.6 (3)
O7—Ho1—N1—C2	101.9 (3)	O8—Ho1—O3—C7	116.6 (3)
O1—Ho1—N1—C2	−0.2 (3)	N1—Ho1—O3—C7	4.5 (3)
O2^ii^—Ho1—N1—C2	−48.1 (3)	C5—C4—O5—Ho1^iv^	−106.4 (4)
O8—Ho1—N1—C2	42.8 (3)	C3—C4—O5—Ho1^iv^	73.3 (4)

Symmetry codes: (i) *x*−1/2, −*y*+3/2, *z*−1/2; (ii) −*x*+3/2, *y*+1/2, −*z*+3/2; (iii) −*x*+3/2, *y*−1/2, −*z*+3/2; (iv) *x*+1/2, −*y*+3/2, *z*+1/2.

### Hydrogen-bond geometry (Å, °)

**Table:**

*D*—H···*A*	*D*—H	H···*A*	*D*···*A*	*D*—H···*A*
O9—H8W···O2^v^	0.86 (1)	2.33 (4)	3.080 (5)	145 (6)
O9—H7W···O4	0.86 (1)	1.84 (1)	2.693 (5)	170 (5)
O8—H6W···O9^vi^	0.86 (1)	2.28 (3)	3.026 (5)	145 (5)
O8—H5W···O9^vii^	0.86 (1)	1.84 (2)	2.689 (5)	168 (5)
O7—H4W···O3^vii^	0.86 (1)	2.08 (3)	2.788 (4)	139 (4)
O7—H3W···O5^viii^	0.87 (1)	1.92 (3)	2.711 (4)	152 (4)
O6—H2W···O1^ii^	0.86 (1)	1.85 (2)	2.671 (4)	159 (5)
O6—H1W···O4^ix^	0.86 (1)	1.83 (1)	2.687 (4)	171 (5)

Symmetry codes: (v) *x*−1, *y*, *z*; (vi) *x*+1/2, −*y*+3/2, *z*−1/2; (vii) −*x*+1/2, *y*−1/2, −*z*+3/2; (viii) −*x*+1, −*y*+1, −*z*+2; (ii) −*x*+3/2, *y*+1/2, −*z*+3/2; (ix) −*x*+1, −*y*+2, −*z*+2.
